# Subtle Cutback Management and Exhaustion: Qualitative Job Insecurity as a Mediator in a Sample of Dutch and Belgian Employees

**DOI:** 10.3390/ijerph20095684

**Published:** 2023-04-28

**Authors:** Yvette Akkermans, Dave Stynen

**Affiliations:** Faculty of Management, Open Universiteit, 6419 AT Heerlen, The Netherlands; y_akkermans@hotmail.com

**Keywords:** cutback measures, qualitative job insecurity, exhaustion

## Abstract

As an answer to crises such as COVID-19, organizations implemented more subtle forms of cutback measures such as wage moderation, loan sacrifice and recruitment freezes aimed at maintaining a financially healthy organization. In this study, the association between subtle cutback management and employee exhaustion was studied, and it was investigated whether this potential linkage can be explained by employee perceptions of increased qualitative job insecurity or the fear that valued features of the job will decrease in the near future. This research thereby extends prior research on the consequences of cutback management as well as regarding the antecedents of qualitative job insecurity. A cross-sectional online survey was conducted on a sample of workers (N = 218) active in various organizations in the Netherlands and Belgium. Regression analysis was applied to test hypotheses. Mediation was investigated by means of Hayes PROCESS macro. The results of the study indicate that there is no direct relationship between subtle cutback measures deployed at the workplace and employee exhaustion. However, the analyses further reveal that subtle cutback management is positively related to the experience of qualitative job insecurity in workers and that enhanced qualitative job insecurity is positively related to employee exhaustion. Qualitative job insecurity fully mediates the relationship between subtle cutback management and employee exhaustion.

## 1. Introduction

According to the Dutch Social and Economic Council report of June 2021, the Dutch society and economy, like many other Western countries, face major challenges such as transitions in the areas of digitization and energy production, in addition to already longstanding issues such as globalization and an aging population. Major shifts in employment across sectors and changes in the nature and content of work are a result of these ongoing changes [1]. The worldwide COVID-19 pandemic added significant challenges, including the companies’ survival [2]. Lockdowns and closure of “non-essential” companies during several lengthy periods in 2020 and 2021 struck companies and their businesses hard. In this setting, it is no surprise that the COVID-19 pandemic has directly affected physical and mental health worldwide [3]. There is also evidence that COVID-19 has led to deteriorating working conditions for many employees, including involuntary work from home, social distancing, disengagement, inequality and layoffs [4]. COVID-19 has contributed to a higher risk of employees encountering burnout [4]. Burnout is known to relate to negative outcomes such as sickness absence [5], and earlier research has indicated that the length of sick absence due to mental disorders predicts disability retirement more strongly than any other diagnosis [6]. In 2021, organizations in the Netherlands faced the highest sickness absenteeism rate in 17 years. The combined category “psychological issues, stress and burnout” was the fourth most important reason for sick leave as found by Statistics Netherlands [7]. Burnout prevails in 16% of the Dutch population, based on 2021 data processed by the Dutch Organization for Applied Scientific Research [8].

Throughout the COVID-19 pandemic, the Dutch government introduced various financial support packages and relaxed taxation rules for companies and self-employed workers to survive financially [9]. Despite this governmental financial support, companies are going through continuous organizational changes to remain competitive in such times of transition and economic crisis [10]. A particular type of ongoing organizational change is a result of cutback management. Levine (1979, p. 180) described cutback management as “organizational change toward lower levels of resource consumption” [11]. Cutback strategies are implemented to deal with financial decline and include more extreme forms of change such as downsizing, as well as more subtle forms such as wage moderation, recruitment freezes, reduced service provision, or merging of teams [12]. From the start of the pandemic in the Netherlands, various companies were forced to implement extreme and/or more subtle cutback measures to stay financially healthy [13,14]. The Dutch airline company KLM announced a reduction of 4500 to 5000 full-time equivalents (−15%), despite a governmental support package of EUR 4.4 billion [15]. At the same time, KLM announced a salary freeze; the agreed 2.5% salary increase was not implemented in 2021, and there is a wage moderation policy for the coming 6 years [16]. In addition, approximately 3000 pilots were asked for a loan sacrifice of 20% during the duration of the support package. Other examples of companies in the Netherlands that announced the implementation of cutback measures to survive and deal with financial turmoil are Dutch bank ING, with a reduction of 1000 FTEs worldwide [17], Dutch Railway company (NS), which announced a reduction of 2300 jobs in the coming years and a loan sacrifice of 10% by top management [18], and Holland Casino, announcing a reorganization with forced redundancies [19]. 

The aim of implementing cutback measures is to survive financial decline by reducing expenditures or costs in a way that business can be continued [12]. However, the pursuit of corporate financial health by implementing cutback measures may come with a hidden “cost”, as it may have implied unintended negative health effects for employees. Cutback management can be considered as organizational changes [20] and can, in line with the Job Demands-Resources model (JD-R model), be considered as job demands leading to burnout in workers in particular when insufficiently compensated by resources [21]. The association between the more extreme forms of cutback measures, such as downsizing, and psychological ill-health is known to be positive [22,23,24,25]. However, concerning the more subtle forms such as wage moderation, recruitment freezes, reduced service provision and merging of teams, the consequences for employees are less clear-cut [12]. Thus far, little is known whether there is a relation between these more subtle cutback measures and the risk of burnout in employees. Therefore, the purpose of this study was to investigate the relationship between implementation of more subtle forms of cutback measures and exhaustion, which is the core component of burnout [26]. It is important to study this relationship, since this topic has not received much attention in the literature and because such measures are a fairly new phenomenon and have been applied on a larger scale by organizations to tackle contemporary crises such as the COVID-19 pandemic or energy crisis. 

In addition to studying the relationship between subtle cutback management and exhaustion, it is important to be able to explain this possible relationship. The risks of these organizational changes and the emerging flexibilization most often end up faced by workers, who experience a loss of security regarding either the continuity or quality of their jobs as a result [1,27]. This is what is called in the literature “perceived job insecurity” [24]. Previous research has shown that job insecurity impedes workers’ health and well-being [27,28,29,30]. Consequences of job insecurity have been a subject of previous studies; this research adds to the knowledge on the organizational determinants of job insecurity, to which less attention has been paid [27], especially when it concerns qualitative job insecurity. Cutback management may represent a particular type of ongoing organizational change in the context of financially troubled organizations [11] and hence may lead to an increase in job insecurity, which in its turn may lead to impaired health and well-being indicated by more mental distress and burnout [27]. 

Greenhalgh and Rosenblatt already stated in 1984 that the loss of valued job features, instead of losing a job itself, is an important, but often overlooked, aspect of job insecurity [30]. This is what Ashford, Lee and Bobko called “qualitative job insecurity” (threats to valued job features) instead of threats to the job itself, which is referred to as “quantitative job insecurity” [31]. This study explores the relationship between subtle forms of cutback measures and exhaustion and whether this relationship can be explained or mediated by the experience of qualitative job insecurity. The more subtle cutback measures are likely to change valuable features of the job such as, for example, the employment relationship, working conditions, wage and promotion possibilities [32,33]. Therefore, this study connects the organizational change and well-being literature by investigating qualitative job insecurity as a mediating mechanism. Understanding the hidden costs of subtle cutback management, such as employee ill-health, and the processes that can explain these are valuable because implementing more subtle cutback measures to deal with financial decline is currently applied to cope with transitions and times of hardship [2]. In sum, the overall research question that is addressed in this research is: “To what extent is subtle cutback management related to employee exhaustion, and is this relationship mediated by perceptions of qualitative job insecurity”. In what follows, we first elaborate on the key concepts and theoretically underpin the suggested relationships. 

### 1.1. Exhaustion

Schaufeli and Buunk (1996, p. 311) used a metaphor for burnout: “a state or process of exhaustion similar to the smothering of a fire or the extinguishing of a candle” [34]. This research defines burnout as Maslach, Schaufeli and Leiter (2001, p. 398) [26] did: “a syndrome of chronic exhaustion, a cynical, negative attitude regarding work, and reduced professional efficacy that could occur in any job”. In line with this definition, three major dimensions can be extracted that capture the burnout phenomenon: exhaustion, feelings of cynicism and feelings of inefficacy [35]. Exhaustion is known as a response to exposure to stressors and is considered the central component of burnout [26]. 

The JD-R model is a comprehensive model to understand how job stressors can cause burnout. Demerouti and colleagues showed in their seminal work that high job demands, particularly in combination with low job resources, are the most important antecedents of exhaustion [21]. Whereas job demands are defined by the latter authors (p. 501) as “those physical, psychological, social, or organizational aspects of the job that require sustained physical and/or psychological costs”, job resources can be defined as “those physical, psychological, social, or organizational aspects of the job such as social support or job autonomy that are functional in achieving work goals, reduce job demands and the associated physiological and psychological costs and stimulate personal growth and development”. Schaufeli (2017, p. 121) called job demands “the bad things at work that drain energy, such as work-overload, conflicts with others and future job insecurity” [5]. Job demands comprise job characteristics such as task interruptions, workload, and work home interference, but may also encompass demanding characteristics of the broader working environment, such as organizational changes, that go beyond the immediate job level [20]. Following the JD-R model, it is argued that when job demands are high and are not compensated by job resources [21], the employee’s energy diminishes and can result in exhaustion [5]. This is substantiated in meta-analytical reviews [36,37] in which the relation between high demands, low resources and burnout was found. In particular, high job demands were identified as having the strongest relation with exhaustion [37].

### 1.2. Subtle Cutback Management and Exhaustion

One of the earliest definitions of cutback management is described by Levine (1979, p. 180) as managing organizational change toward lower levels of resource consumption and organizational activity [11]. Subtle cutback management refers, in contrast to its more severe counterparts such as forced redundancies, to measures such as wage moderation, recruitment freezes, reduced service provision and merging of teams with the aim to accommodate organizational financial decline [12]. It can be argued that organizational changes can be regarded as a job demand [20]. From the JD-R model, it is known that the employee’s energy leaks away when job demands are high and insufficiently compensated by job resources. Empirically, clear associations were found between major organizational changes such as restructuring, downsizing and outsourcing and health complaints [32,38,39]. Downsizing can, therefore, be identified as a risk factor for the health of employees [39,40]. 

Alternative theoretical frameworks, such as the psychological contract theory, would lead to similar expectations. Rousseau (1990, p. 9) defines a psychological contract as “individual beliefs, shaped by the organization, regarding an exchange agreement between individuals and their organization” [41]. A breach of the psychological contract implies that employees feel that their employer fails to fulfil one or more of their promised obligations [42]. Pate and colleagues have used this notion in their process model on how employees exposed to organizational changes such as cost reduction could experience psychological contract breach, leading in turn to unfavorable outcomes in workers [43]. Organizational changes such as cost-cutting initiatives can be viewed as a breach of promises made by organizations to employees concerning the availability of resources at work [44]. In several studies [45,46,47,48], positive associations between psychological contract breach and mental health complaints such as burnout and general work stress were found. Based on premises of the JD-R model and the psychological contract theory, we hypothesize that:

**H1.**  *Subtle cutback management is positively related to exhaustion.*

### 1.3. Qualitative Job Insecurity and Exhaustion

Greenhalgh and Rosenblatt (1984, p. 438) define job insecurity as “perceived powerlessness to maintain desired continuity in a threatened job situation” [30]. They emphasized that insecurity perceptions are largely driven by the threat of job loss, the desire for continuity in employment, the risk of losing desirable job features, and the powerlessness to be able to do anything to change the situation [30,49]. Phenomena described by Greenhalgh and Rosenblatt [30] leading to job insecurity are often forced redundancies or downsizing, which are severe cutback measures threatening the job itself. This is what Ashford, Lee and Bobko (1989) called “quantitative job insecurity” [31]. Besides the concerns about the continuation of the job itself, Greenhalgh and Rosenblatt stated in their seminal article in 1984 that the loss of valued job features is an important but often overlooked aspect of job insecurity [30]. This threat to valued job features is what Ashford, Lee and Bobko called “qualitative job insecurity” [31]. Examples of valued job features are employment relationships, working conditions, career prospects and salary development [32,33]. Vander Elst et al. [33] framed qualitative job insecurity as a work stressor which would, in line with the JD-R model [21], imply physical and/or psychological costs and hence would be associated with ill-health, including employee exhaustion. Additionally, earlier empirical research has shown an association between job insecurity and exhaustion [29,50,51,52,53,54]. Therefore, we hypothesize:

**H2.**  *Qualitative job insecurity is positively related to exhaustion*.

### 1.4. Subtle Cutback Management and Qualitative Job Insecurity

Organizational changes may also alter the psychosocial working environment of employees, including aspects such as the amount of work and role conflict [40,55]. Obviously, organizational changes naturally coincide with a degree of unpredictability, but it is also likely that changes may evoke specific feelings of immediate job unpredictability as well as harm long-term employment prospects [25]. A theoretical underpinning can be found in Rafferty and Griffin’s three-dimensional model of employees’ perception of organizational change [56]. In this model, which is founded on the cognitive appraisal theory of Lazarus and Folkman [57], it is suggested that organizational changes may be appraised as more threatening when the frequency of changes is higher, the anticipated impact of the change is stronger, and the change is experienced as unplanned. Hence, the more subtle cutback measures are deployed, the more employees may feel threatened. As subtle cutback management is often executed because of poor organizational performance or organizational decline [49] and is focused on reducing resource deployment, it has the potential of being perceived by employees as impacting their jobs in a negative way. The appraisals of threats to one’s job gives rise to feelings of job insecurity [58]. Empirically, research has also established linkages between organizational change, low organizational performance and enhanced feelings of quantitative job insecurity [27,59,60,61]. As subtle cutback management is not directed to layoffs, but rather changes in roles and structures from a cost-cutting point of view, we anticipate that appraisals of threat are reflected in feelings of qualitative job insecurity. Hence, we hypothesize:

**H3.**  *Subtle cutback management is positively related to qualitative job insecurity.*

Given the aforementioned reasoning, which suggests mediation, we also formally hypothesize:

**H4.**  *Qualitative job insecurity mediates the positive relationship between subtle cutback management and exhaustion.*

**Figure 1:**
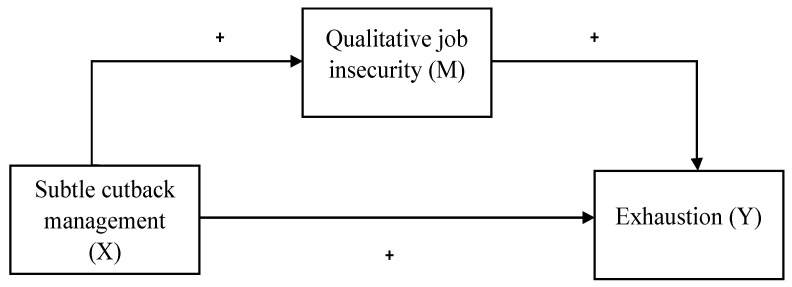
Conceptual model.

Our conceptual model is depicted in Figure 1. 

## 2. Materials and Methods

### 2.1. Participants and Procedure

We used data from a cross-sectional structured online survey conducted in March and April 2022 among Dutch-speaking employees. Participants were recruited in the network of the researchers via social media platforms including LinkedIn, Instagram and Facebook. Respondents were encouraged to share the announcement within their networks. Eligible respondents were employees, living and working in the Netherlands or Belgium, aged 18 to 67. Potential interested participants could indicate their willingness to participate in the study by contacting the researchers on a specific research email address. The link to participate in the survey was then sent to the email address of the participant and not shared directly on social media for purposes of data protection. Attached to the email was a letter provided by the researchers, covering all aspects of the research. Online informed consent was first obtained from all participants before participating in the survey. The online questionnaire was conducted via LimeSurvey and was open for three weeks. A reminder was sent after two weeks.

The initial number of respondents was 271. Based on guidelines [62], respondents with more than 10% missing values (N = 30) were deleted in this sample after the missing value analysis. The remaining 241 respondents were checked on age (between 18 and 67 years old) and employment inclusion criteria (“currently employed by an employer”). Every respondent not matching these criteria was deleted. This resulted in the exclusion of another 23 respondents (1 “unemployed”, 20 “fully self-employed”, 1 “incapacitated not employed by an employer” and 1 “employed by an employer but with a self-employment contract”). The number of eligible respondents in the sample was 218, all of whom were employees with an employment contract.

Characteristics of the employees in the sample (N = 218) can be found in Table 1. Within the sample, 41.3% were male, and 58.7% female. The average age was 40.0 years. Of the respondents, 11% had secondary school as their highest degree, 21.1% intermediate vocational education, 46.8% had a bachelor’s degree, and 21.1% a master’s degree. Of all respondents, 37.2% worked in the public sector, and 62.8% in the private sector. Most of the respondents (63.8%) worked in bigger organizations with more than 250 employees, 21.6% in mid-sized organizations (51–250 employees), 9.6% in a small organization (11–50 employees), and 5% worked in a micro company with less than 10 employees. All respondents were employees, of which most had an indefinite contract (81.7%), and 15.6% had a contract for a definite period. Of the respondents with an indefinite or definite employment contract, 2.3% both had a contract with an employer and were also partially self-employed at the time of the survey. One respondent (0.5%) indicated “no contract” but had employment status “working, employed by employer” and was, therefore, included in the sample. A small number of respondents had flexible contracts: 1.8% had a temporary agency contract, and 0.9% had an on-call contract. 

### 2.2. Measures

#### 2.2.1. Exhaustion

Exhaustion was measured with the exhaustion dimension scale of the Oldenburg Burnout Inventory (OLBI). The OLBI exhaustion dimension covers affective, physical and cognitive aspects of exhaustion [63]. The OLBI has been validated in various studies [21,64] and over a broad range of occupational groups [63]. Since the population of this study was diverse and not focused on one industry or functional group, the OLBI seemed a suitable instrument to assess exhaustion. The OLBI exhaustion dimension consists of eight items (e.g., “There are days when I feel tired before I arrive at work”). The scale includes four positively worded items and four negatively worded items. Participants were asked to respond to the items by using a 5-point scale ranging from 1 (“strongly disagree”) to 5 (“strongly agree”). The negatively worded items were recoded so that high scores referred to high levels of exhaustion [63]. Cronbach’s α for exhaustion was 0.88. As exhaustion is conceptually characterized by affective, physical and cognitive aspects, and principal component analysis (PCA) revealed one underlying factor (see Section 2.2.5), an overall scale score was computed for every employee. 

#### 2.2.2. Subtle Cutback Management

A formative measurement approach was used to assess subtle cutback management. A formative measure provides a means of measuring a construct from a diverse and potentially disparate set of observable phenomena that may not necessarily be correlated [65,66]. Different cutback measures are specific and actionable attributes of a phenomenon to which workers may have been exposed [12], which does not tie in with a reflective measurement approach. The indices were developed based on both the definition of cutback management and the related measures that were described in the literature [12] or were based on examples in recent news articles that fitted with the definition. The question was: “Has your organization taken one or more of the following cutback measures in the past year with the purpose to reduce costs?” (Answer choices: “yes”/“no”). Each of the nine presented types reflected one possible cutback measure, including: “Recruitment freeze”; “Merger of teams or departments within the organization”; “Reduction of management layers in the organization”; “Voluntary redundancies”; “Changes to the employment benefits like working hours within the organization”; “Major changes to the offered products and services of the organization”; “Reduced service provision”; “Wage moderation”; “Loan sacrifice”. The items were translated by the researcher and via back-translation checked by a native English speaker. An overall index, “subtle cutback measures”, was created for these dichotomous items by summing all “yes” answers. This resulted in a range from 0 to 9 points. A higher index value indicated that the respondent experienced more subtle cutback measures within the organization.

#### 2.2.3. Qualitative Job Insecurity

Qualitative job insecurity was measured with the Multidimensional Qualitative Job Insecurity scale (MQJIS) [67]. The MQJIS consists of eight items that express feelings of insecurity regarding four dimensions: social relationships, employment conditions, working conditions and work content. These four dimensions fit with the categorization of job stressors at the workplace, by Le Blanc and colleagues [68], as either tied to the content of work (e.g., the complexity, variation), the working conditions (e.g., noise, degree of physical demands), employment conditions (e.g., career prospects) or social relationships (e.g., social support). Example items of these dimensions were respectively: “I feel insecure about the future content of my job”, “Chances are my workload will increase in the future”, “I am insecure about my chances of promotion” and “I am not sure which colleagues I will be soon cooperating with”. Responses were given on a 7-point Likert scale, from 1 = “Not at all true to me” to 7 = “Completely true to me”. The Cronbach’s α for qualitative job insecurity was 0.75. According to the literature, the instrument can be used to measure qualitative job insecurity as a single construct as a measurement model comprising four first-order factors (dimensions), and one second-order factor (general qualitative job insecurity) fitted the data best [67]. Also in our study, PCA indicated the importance of one underlying factor (see Section 2.2.5). Hence, we used the scale to measure overall feelings of qualitative job insecurity. 

#### 2.2.4. Control Variables

To draw more definite conclusions, possible confounders were included in the study [69]. Based on earlier research, the demographic variables “gender”, “age”, “education”, “size”, “sector” and “contract status” were interesting to be included in this research as control variables. Sector, contract type, age, gender and education have been identified as antecedents of qualitative job insecurity [27]. “Size” was included since it has been identified as a possible predictor for threats to job security [70]. “Forced redundancies” was added as a control variable because subtle cutback measures may coincide with the more severe measures. 

“Gender” was measured with the question “what is your gender?”, with three answers (“male”/“female”/“non-binary”). The demographic variable “age” was measured by asking “what is your age (in years)?” Respondents were also asked the question “in which sector do you work?”, with two possible answers: “public” or “private” sector. The question to measure “education” was “what is your highest obtained degree?”, with seven different answers (e.g., “master’s degree”). “Size” was measured with the question “what is the size of the organization you are working for?”, and respondents could choose between four categories (e.g., “51–250 employees”). The variable “contract status” was measured with the question “which type of contract do you have?”, and six response options (or a combination of these six options) were possible (e.g., “contract for a definite period”). Respondents were asked whether they have experienced “forced redundancies” in their organization during the past year with the purpose of reducing costs, with two answer possibilities (“yes”/“no”).

To perform the hierarchical multiple regression analyses, dummy variables were created for the control variables. “Contract status” was controlled for by separating two groups: all the flexible contract options (definite contract, on-call contract, agency contract and self-employed) were clustered as “flexible contracts”, and the second category was “indefinite contracts”. “Flexible contracts” was used as a reference category. The respondent with “no contract” status was coded as a missing value. “Size organization” was controlled for by distinguishing three groups: the two categories “0–10 employees” and “11–50 employees” were grouped together as “size organization ≤50 employees”, and the other two groups were “size organization 51–250 employees” and “size organization more than 250 employees”. “Size organization ≤50 employees” was used as a reference category. “Forced redundancies” was controlled for, with “no forced redundancies” as reference category. 

#### 2.2.5. Data Analyses

The analysis of the data was conducted via SPSS, version 28. For the mediation analyses, the Hayes PROCESS macro version 4.1 was added to SPSS, version 28. 

First, descriptive statistics were computed for all variables (mean and standard deviation), reliability if relevant, and all intercorrelations among the variables. The reliability of the variables was verified by analyzing the Cronbach’s α. An α > 0.70 was considered sufficient [71]. Although validated scales were used in this research, exhaustion and qualitative job insecurity were subjected to a principal component analysis (PCA) to evaluate whether the data may, as expected, be summarized on a single summed measure prior to the hierarchical multiple regression analysis in this research [72]. Subtle cutback measures were excluded from the PCA, since it concerned a formative measure [66]. 

The data were assessed as suitable for a PCA. Since evaluation of the correlation matrices of the items revealed the presence of many coefficients of 0.30 and above, the sample size was sufficient (above N = 150) and the Kaiser–Meyer–Olkin measure of sampling adequacy (KMO) value exceeded the required value of 0.60, with a value of 0.90 for exhaustion and 0.78 for qualitative job insecurity, and the Bartlett’s test for both variables was significant (*p* ≤ 0.001) [72]. For exhaustion, the principal component analysis revealed one component with an eigenvalue above 1, explaining 54.2% of the variance, and all of the items of the exhaustion scale loaded substantially on this factor. For qualitative job insecurity, the principal component analysis showed that three components had an eigenvalue above 1 and explained 38.34%, 14.08% and 12.57% of the variance, respectively. All items loaded substantially on the first factor (range 0.17–0.74), which had an eigenvalue of 3.07. The other two factors had marginal eigenvalues of 1.13 and 1.01 and reflected residual commonalities in social relationships or employment conditions, respectively. Since a clear general factor was found, and all items were theoretically relevant to the scale and the Cronbach’s alpha was acceptable, no adaptions were made to the scale. 

Hierarchical multiple regression analyses were conducted to test our hypotheses. Because “age” and, after recoding, “size organization 51–250 employees”, “size organization more than 250 employees”, “forced redundancies” and “indefinite contracts” correlated significantly with the dependent or independent variables, these were all included as control variables in the first step of the regression analysis. In the second step of the regression analysis, we added the explanatory independent variable subtle cutback management to explain exhaustion (model 3); qualitative job insecurity to explain exhaustion (model 2) and subtle cutback management to explain qualitative job insecurity (model 1). 

Finally, the Hayes PROCESS macro was executed [73]. This concerns a method to analyze the effect that variable X (in this research, subtle cutback management) has on variable Y (exhaustion) through an intervening variable M, a mediator, in this case qualitative job insecurity, depicted in Figure 1. Using this method, the direct effect of X (subtle cutback management) on Y (exhaustion) was quantified as well as the total indirect effect of X on Y through M (qualitative job insecurity) [73]. The number of bootstrap samples used for percentile bootstrap confidence intervals was 1000; this was considered sufficient given the 95% confidence intervals [74]. 

## 3. Results

The Pearson correlation analysis, of which the results are summarized in Table 2, showed no significant correlation between subtle cutback management and exhaustion (r = 0.09, *p* = 0.19). There was a positive correlation between qualitative job insecurity and exhaustion (r = 0.32, *p* < 0.001) and a positive correlation between subtle cutback management and qualitative job insecurity (r = 0.25, *p* < 0.001). Concerning the demographic variables, only “contract status” correlated statistically significantly with exhaustion (r = 0.16, *p* = 0.02). Qualitative job insecurity and age showed a negative correlation (r = −0.30, *p* < 0.001), and subtle cutback management and the size of the organization showed a positive correlation (r = 0.22, *p* < 0.001). 

**Figure 2:**
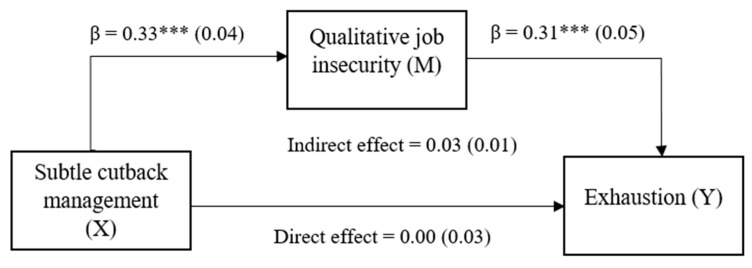
Mediation of the effect of subtle cutback management on exhaustion (total effect: 0.04, 95% CI = −0.02, 0.09) by qualitative job insecurity (indirect effect: 0.03, 95% CI = 0.01, 0.06). *** *p* < 0.001.

The results of the regression analyses are depicted in Table 3. The regression analyses (Model 3) showed no support for hypothesis 1 since there was no significant association between subtle cutback measures and exhaustion (β = 0.12, *p* = 0.14). The results (Model 2) further showed that qualitative job insecurity was positively related to exhaustion (β = 0.31, *p* < 0.001), corroborating hypothesis 2. In line with hypothesis 3, subtle cutback management was positively related to qualitative job insecurity (β = 0.33, *p* < 0.001), as could be seen in Model 1. Finally, the Hayes mediation analysis, depicted in Figure 2, revealed that the indirect effect of subtle cutback management on exhaustion via the mediator qualitative job insecurity was significant: 0.03 with a 95% confidence interval that ranged between the bootstrapped lower level of 0.0105 and the upper level of 0.0576. The indirect effect was significant, as the value of zero was not in the confidence interval [75]. Hence, support was found for mediation, in line with hypothesis 4.

Overall, these findings suggested full mediation, since there was no significant direct association (see Figure 2) between cutback management and exhaustion but an indirect-only effect via the mediator qualitative job insecurity.

## 4. Discussion

The pursuit of a financially healthy organization in implementing more subtle forms of cutback measures might, as expected, come at a cost, as it may lead to an unintended negative health effect for employees. Implementing subtle forms of cutback measures showed an association with perceived threats to valued job features (qualitative job insecurity), and qualitative job insecurity was, in turn, related to exhaustion among employees. Although subtle cutback management was not directly related with employee exhaustion, support was found for an indirect path through qualitative job insecurity. Thereby, this study adds to the organizational change and well-being literature, first by focusing on subtle instead of extreme cutback measures, and second by paying attention to the qualitative aspect of job insecurity as a mediating mechanism in the relation between subtle forms of cutback management and exhaustion.

With regard to cutback management, the expectation in this study was that subtle forms of cutback management were directly and positively related to exhaustion. We expected that such measures were considered organizational changes [11] that would be experienced by workers as demanding to deal with [20]. In line with the Job Demands-Resources (JD-R) model, such job demands would lead to energy depletion [5]. In addition, in the organizational change literature [43], another explanation was found in psychological contract theory. Organizational changes, in which cost reductions are explicitly mentioned, can be conceived as a contextual factor that could lead to perceptions of breach of the psychological contract that employees have with their employers. Such breaches are presumed to have negative implications for employee well-being. Subtle cutback management measures were, however, not directly related to employee exhaustion. This is different compared to studies about the more extreme forms of cutback measures, such as downsizing, that established effects on psychological ill-health [22,23,24,25]. Various reasons could be given for this finding. First of all, not all subtle cutback measures may be seen as demanding. Potentially, measures that imply reductions in the level of service provision may also reduce work pressure, or perhaps measures such as major changes to the offered products and services of the organization may be perceived as challenging existing work procedures that yield opportunities for improvements in job functioning or job crafting at the same time. These concern so-called “challenge stressors”, which may not, per se, have negative health implications, as is the case for “hindrance stressors” [76]. Moreover, theoretically, effects are likely to depend upon levels of experienced job resources [21]. Secondly, it is likely that some measures, such as loan sacrifice, have more potential for psychological contract breach than other measures, such as the reduction of management layers in the organization. Hence, subtle cutback management may not uniformly trigger the presumed processes central in the JD-R model or psychological contract theory. 

Although no direct association was found between subtle cutback management and exhaustion, an indirect association was found via employee perceptions of qualitative job insecurity. First of all, this suggests that such measures may bring uncertainty about the quality of the job in the near future. This is a valuable addition to the organizational change literature, as we demonstrated that organizational changes such as subtle cutback management may alter the psychosocial working environment. Rafferty and Griffin’s three-dimensional model of employees’ perception of organizational change [56] may explain why these circumstances could lead to enhanced feelings job insecurity [58]. We thereby also add to research on the organizational determinants of job insecurity, which has received less attention compared to research on the consequences of job insecurity [27]. However, to what extent these changes were planned, which constitutes the third dimension of Rafferty and Griffin’s model [56], was not assessed in this study. Secondly, in line with earlier studies [50], our study found support for the negative association of qualitive job insecurity and exhaustion, as was expected based on the JD-R model [21]. Qualitative job insecurity has been given less attention compared to its quantitative counterpart. Our study further adds insight into the factors that could contribute to exhaustion, but not in the broader construct of “burnout” that comprises three components: exhaustion, feelings of cynicism and feelings of inefficacy [35].

This study has several limitations. First of all, because of the cross-sectional design of the study, no cause–effect relationships could be inferred. Secondly, due to self-selected sampling techniques, it is possible that employees who experienced more organizational changes may have been more eager to respond to our survey. Hence, findings cannot be generalized to the population. Related to this, in our sample we had an overrepresentation of highly educated employees, of which a large majority also held permanent contracts and worked for major organizations. Confounders explained little variance in our outcome variables, maybe because variation in these characteristics was limited. It appeared that levels of qualitative job insecurity were lower at older age. Potentially, older workers are more spared of organizational changes such as cutback management, or maybe they see more opportunities to deal (i.e., craft their jobs) with these changes [76]. The finding that exhaustion was higher among employees with an indefinite contract could have been caused by exposure to higher workloads and responsibilities compared to employees with more flexible contracts. Thirdly, the variable “subtle cutback management” was measured as an index score out of nine possible forms of subtle cutback measures. Although based on the literature and attuned to recent developments, the list was probably not exhaustive. On average, our sample seemed to have been exposed to lower levels of subtle cutback measures, and it remains difficult to compare levels of exposure in this study with those of other sources. In this context, the measurement may be prone to recall bias (“past year”) and common-method bias, as all measures are self-reported. On the positive side, we took confounders into account, and our results cannot be explained by the association of subtle cutback management with more severe forms of forced redundancies. As we controlled for such influences, our results suggest that subtle cutback management plays a unique role in explaining variation in employee exhaustion and qualitative job insecurity. 

### Recommendations for Practitioners and Future Research

Implementing subtle cutback measures fits with organizations’ aims of remaining financially competitive in challenging times. Nevertheless, our study indicates that even the more subtle forms of cost reduction initiatives may have unintended negative effects on employee well-being. From that point of view, organizations can be advised to turn away from such unsustainable practices or at least to take adversity into account when considering such measures. However, cutback measures are, in certain situations, unavoidable to anticipate to changes. What is crucial in such situations is that associated feelings of uncertainty should be mitigated. A key finding is that negative health consequences seem to manifest themselves in so far that subtle cutback management succeeds in triggering feelings of qualitative job insecurity. The literature provides ways to reduce feelings of job insecurity or to buffer the consequences of job insecurity: e.g., by enhancing transparent and open communication regarding changes, employee participation in decision making, procedural justice, or the employability of employees [77,78]. Following Rafferty and Griffin [56], supportive leadership makes a difference in the change perceptions of workers in general. Their findings show that employees who experienced supportive leadership reported less psychological uncertainty. A supportive leader provides information and advice that an employee can rely upon when confronted with change [56]. Again, open and transparent communication is key. From the perspective of the JD-R model, leaders should strive to establish balance between job demands and job resources during times of organizational change when the demands can be experienced as higher. This risk can be alleviated by creating sufficient resources such as social support from co-workers, leaders and the organization.

The results provide interesting and potentially new avenues for future research. The first recommendation is to study these relationships over time. In addition, we only investigated one facet of burnout; other components of burnout or indicators of well-being such as work engagement could be of interest. Concerning mediation, alternative pathways such as psychological contract breach, but also quantitative job insecurity or other psychosocial variables that fit in the context of organizational change, such as role conflict and role ambiguity, are of interest. Regarding subtle cutback management, it could be argued that new ways of measurement can be developed to capture the extent to which such changes are experienced as planned and are also experienced as threatening versus challenging. Finally, in particular, job resources or good practices in change management (e.g., communication) can be studied as moderators that play a buffering role in the unfolding processes. 

## Figures and Tables

**Table 1:** Characteristics of the respondents (N = 218).

	%
**Gender**	
Male	41.3
Female	58.7
Non-binary	0
**Age in years**	
60–67	4.1
50–59	22.5
40–49	21.1
30–39	28.0
18–29	22.5
Missing	1.8
**Education**	
No diploma	0
Primary school	0
High School	11
Intermediate vocational degree	21.1
Bachelor’s degree	46.8
Master’s degree	21.1
Doctorate/PhD	0
**Sector**	
Public	37.2
Private	62.8
**Employment status (combinations possible)**	
Incapacitated	0.5
Unemployed	0
Working, self-employed	3.2
Working, employed by an employer	100
Pension	0
**Contract status**	
No contract	0.5
Definite contract (temporary)	15.6
Indefinite contract	81.7
Agency contract	1.8
On-call contract	0.9
Self-employed	2.3
**Size of the organization**	
0–10 employees	5.0
11–50 employees	9.6
51–250 employees	21.6
More than 250 employees	63.8

**Table 2:** Means (M), Standard Deviations (SD), and Correlations (N = 218).

	Variables	M	SD	1	2	3	4	5	6	7	8	9
1.	Exhaustion	2.55	0.71									
2.	Qualitative job insecurity	3.20	1.02	0.32 ***								
3.	Subtle cutback measures	1.40	1.81	0.09	0.25 ***							
4.	Age	39.98	11.77	−0.12	−0.30 ***	0.04						
5.	Gender	1.59	0.49	0.09	0.03	−0.06	−0.10					
6.	Education	4.78	0.90	0.01	−0.05	0.02	−0.04	−0.02				
7.	Sector	1.63	0.48	0.02	−0.08	0.00	−0.06	−0.01	−0.05			
8.	Size organization	3.44	0.86	−0.06	0.02	0.22 ***	0.10	0.01	0.15 *	−0.17 *		
9.	Contract status	2.45	6.62	0.16 *	0.05	0.02	−0.09	0.06	0.10	0.05	0.03	
10.	Forced redundancies	0.14	0.35	0.03	0.01	0.41 ***	−0.10	0.02	0.01	0.18 **	0.08	−0.03

* *p* < 0.05, ** *p* < 0.01, *** *p* < 0.001.

**Table 3:** Regression results for the mediation of qualitative job insecurity between subtle cutback management and exhaustion (N = 218).

	Model 1 Subtle Cutback Management— Qualitative Job Insecurity		Model 2 Qualitative Job Insecurity— Exhaustion		Model 3 Subtle Cutback Management— Exhaustion	
Variables	Step 1	Step 2	Step 1	Step 2	Step 1	Step 2
Age	−0.31 *** (0.01)	−0.33 *** (0.01)	−0.10 (0.00)	−0.01 (0.00)	−0.10 (0.00)	−0.11 (0.00)
Size org 51–250 employees (ref. size org ≤50)	0.10 (0.23)	0.09 (0.22)	0.04 (0.17)	0.01 (0.16)	0.04 (0.17)	0.03 (0.17)
Size org more than 250 employees (ref. size org ≤50)	0.08 (0.20)	−0.00 (0.19)	−0.05 (0.14)	−0.08 (0.13)	−0.05 (0.14)	−0.08 (0.14)
Indefinite contracts (ref. flexible contracts)	0.04 (0.01)	0.03 (0.01)	0.17 * (0.01)	0.16 * (0.01)	0.17 * (0.01)	0.16 * (0.01)
Forced redundancies	−0.00 (0.20)	−0.14 (0.21)	0.04 (0.14)	0.04 (0.14)	0.04 (0.14)	−0.01 (0.15)
Subtle cutback management		0.33 *** (0.04)				0.12 (0.03)
Qualitative job insecurity				0.31 *** (0.05)		
Adjusted R^2^	0.08	0.16	0.03	0.11	0.03	0.03
F	4.59 ***	7.66 ***	2.11	5.26 ***	2.11	2.14

Notes: N = 218. Standardized betas are reported, standard error is shown in parentheses. * *p* < 0.05, *** *p* < 0.001.

## Data Availability

The data can be made available on request to the corresponding author.

## References

[1] Dutch Social and Economic Council (2021). Sociaal Economisch Beleid (2021–2025) Zekerheid voor Mensen, een Wendbare Economie en Herstel van de Samenleving. [Social and Economic Policy (2021–2025) Security for People, a Flexible Economy and Recovery of Society].

[2] OECD (2020). Policy Responses to Coronavirus (COVID-19). The Territorial Impact of COVID-19: Managing the Crises Across Levels of Government.

[3] Salameh P., Hajj A., Badro D.A., Selwan C.A., Aoun R., Sacre H. (2020). Mental Health Outcomes of the COVID-19 Pandemic and a Collapsing Economy: Perspectives from a Developing Country. Psychiatry Res..

[4] Kniffin K.M., Narayanan J., Anseel F., Antonakis J., Ashford S.P., Bakker A.B., Bamberger P., Bapuji H., Bhave D.P., Choi V.K. (2021). COVID-19 and the workplace: Implications, issues, and insights for future research and action. Am. Psychol..

[5] Schaufeli W.B. (2017). Applying the Job Demands-Resources model: A ‘how to’ guide to measuring and tackling work engagement and burnout. Organ. Dyn..

[6] Salonen L., Blomgren J., Laaksonen M., Niemelä M. (2018). Sickness absence as a predictor of disability retirement in different occupational classes: A register-based study of a working-age cohort in Finland in 2007–2014. BMJ Open.

[7] Hoogste Ziekteverzuim Werknemers in 17 Jaar [Highest Employee Absenteeism in 17 Years]. https://www.cbs.nl/nl-nl/nieuws/2021/21/hoogste-ziekteverzuim-werknemers-in-17-jaar.

[8] Factsheet Week van de Werkstress 2021 [Factsheet Week of Work Stress]. https://www.monitorarbeid.tno.nl/nl-nl/publicaties/factsheet-week-van-de-werkstress-2021/.

[9] Overzicht Steun- en Herstelpakket [Overview Support and Recovery Package]. https://www.rijksoverheid.nl/onderwerpen/coronavirus-financiele-regelingen/overzicht-financiele-regelingen.

[10] Kiefer T. (2005). Feeling bad: Antecedents and consequences of negative emotions in ongoing change. J. Organ. Behav..

[11] Levine C.H. (1979). More on Cutback Management: Hard Questions for Hard Times. Public Adm. Rev..

[12] Kiefer T., Hartley J., Conway N., Briner R.B. (2014). Feeling the Squeeze: Public Employees’ Experiences of Cutback- and Innovation-Related Organizational Changes Following a National Announcement of Budget Reductions. J. Public Adm. Res. Theory.

[13] Koning P.W.C., Jongen E. (2020). Lessen voor de NOW. (CPB Coronapublicatie).

[14] Tienduizenden banen weg bij Damrakbedrijven [Tens of Thousands of Jobs Lost at Damrak Companies]. https://fd.nl/beurs/1375750/tienduizenden-banen-weg-bij-damrakbedrijven-px38l1caogpfT0.

[15] KLM Past Organisatie Verder aan als Gevolg van de COVID-19-Crisis [KLM Further Adjusts Its Organization as a Result of the COVID-19 Crisis]. https://nieuws.klm.com/klm-past-organisatie-verder-aan-als-gevolg-van-de-covid-19-crisis/.

[16] KLM-Piloten Buigen in Krachtmeting met Hoekstra over Loonoffer [KLM Pilots Weigh in on Wage Sacrifice with Hoekstra]. https://fd.nl/beurs/1363141/ook-piloten-klm-nu-akkoord-met-loonmatiging-over-langere-periode-l1k1car9tvUp.

[17] ING Schrapt Wereldwijd Duizend Banen [ING is Cutting a Thousand Jobs Worldwide]. https://nos.nl/artikel/2355287-ing-schrapt-wereldwijd-duizend-banen.

[18] NS Wil 2300 Banen Schrappen. [NS Wants to Cut 2300 Jobs]. https://fd.nl/ondernemen/1349517/ns-wil-2300-banen-schrappen-qxl1caogpfT0.

[19] Grootste Verlies ooit Voor Holland Casino; 81 Miljoen in Het Rood. [Biggest Loss Ever for Holland Casino; a Loss of 81 Million]. https://nos.nl/artikel/2377453-grootste-verlies-ooit-voor-holland-casino-81-miljoen-in-het-rood.

[20] Van den Broeck A., Vansteenkiste M., De Witte H., Lens W. (2008). Explaining the relationships between job characteristics, burnout, and engagement: The role of basis psychological need satisfaction. Work Stress.

[21] Demerouti E., Bakker A.B., Nachreiner F., Schaufeli W. (2001). The Job Demands–Resources Model of Burnout. J. Appl. Psychol..

[22] Quinlan M., Bohle P. (2009). Overstretched and Unreciprocated Commitment: Reviewing Research on the Occupational Health and Safety Effects of Downsizing and Job Insecurity. Int. J. Health Serv..

[23] Wiezer N., Nielsen K., Pahkin K., Widerszal-Bazyl M., De Jong T., Mattila-Holappa P., Mockałło Z. (2011). Exploring the Link between Restructuring and Employee Well-Being.

[24] Benach J., Vives A., Amable M., Vanroelen C., Tarafa G., Muntaner C. (2014). Precarious Employment: Understanding an Emerging Social Determinant of Health. Annu. Rev. Public Health.

[25] Fløvik L., Knardahl S., Christensen J.O. (2019). Organizational change and employee mental health: A prospective multilevel study of the associations between organizational changes and clinically relevant mental distress. Scand. J. Work. Environ. Health.

[26] Maslach C., Schaufeli W.B., Leiter M.P. (2001). Job Burnout. Annu. Rev. Psychol..

[27] Shoss M.K. (2017). Job Insecurity: An Integrative Review and Agenda for Future Research. J. Manag..

[28] Kalleberg A.L., Vallas S.P. (2018). Precarious Work. Book Series: Research in the Sociology of Work.

[29] Witte H., De Cuyper N., De Handaja Y., Sverke M., Näswall K., Hellgren J. (2010). Associations Between Quantitative and Qualitative Job Insecurity and Well-Being. Int. Stud. Manag. Organ..

[30] Greenhalgh L., Rosenblatt Z. (1984). Job Insecurity: Toward Conceptual Clarity. Acad. Manag. Rev..

[31] Ashford S.J., Lee C., Bobko P. (1989). Content, causes, and consequences of job insecurity: A theory-based measure and substantive test. Acad. Manag. J..

[32] Hellgren J., Sverke M., Isaksson K. (1999). A Two-dimensional Approach to Job Insecurity: Consequences for Employee Attitudes and Well-being. Eur. J. Work. Organ. Psychol..

[33] Vander Elst T., Richter A., Sverke M., Näswall K., De Cuyper N., De Witte H. (2014). Threat of losing valued job features: The role of perceived control in mediating the effect of qualitative job insecurity on job strain and psychological withdrawal. Work Stress.

[34] Schaufeli W.B., Buunk B.P. (1996). Professional Burnout. Handbook of Work and Health Psychology.

[35] Maslach C. (2003). Job burnout: New directions in research and intervention. Curr. Dir. Psychol. Sci..

[36] Lee R.T., Ashforth B.E. (1996). A meta-analytic examination of the correlates of the three dimensions of job burnout. J. Appl. Psychol..

[37] Alarcon G.M. (2011). A meta-analysis of burnout with job demands, resources, and attitudes. J. Vocat. Behav..

[38] Schweiger D., Denisi A. (1991). Communication with employees following a merger: A longitudinal field experiment. Acad. Manag. J..

[39] Vahtera J., Kivimaki M., Pentti J. (1997). Effect of organisational downsizing on health of employees. Lancet.

[40] Kivimaki M., Vahtera J., Pentti J., Thomson L., Griffiths A., Cox T. (2001). Downsizing, changes in work, and self-rated health of employees: A 7-year 3-wave panel study. Anxiety Stress. Coping.

[41] Pearce J.L., Rousseau D.M. (1998). Psychological Contracts in Organizations: Understanding Written and Unwritten Agreements. Adm. Sci. Q..

[42] Robinson S.L., Morrison E.W. (2000). The development of psychological contract breach and violation: A longitudinal study. J. Organ. Behav..

[43] Pate J., Martin G., Staines H. (2000). Exploring the relationship between psychological contracts and organizational change: A process model and case study evidence. Strat. Chang..

[44] Conway N., Kiefer T., Hartley J., Briner R.B. (2014). Doing More with Less? Employee Reactions to Psychological Contract Breach via Target Similarity or Spillover during Public Sector Organizational Change. Br. J. Manag..

[45] Gakovic A., Tetrick L.E. (2003). Psychological contract breach as a source of strain for employees. J. Bus. Psychol..

[46] Jiang L., Probst T.M., Benson W.L. (2015). Organizational context and employee reactions to psychological contract breach: A multilevel test of competing theories. Econ. Ind. Democracy.

[47] Reimann M., Guzy J. (2017). Psychological contract breach and employee health: The relevance of unmet obligations for mental and physical health. Rev. De Psicol. Del Trab. Organ..

[48] Griep Y., Bankins S., Elst T.V., De Witte H. (2021). How psychological contract breach affects long-term mental and physical health: The longitudinal role of effort–reward imbalance. Appl. Psychol. Health Well-Being.

[49] Greenhalgh L., Rosenblatt Z. (2010). Evolution of Research on Job Insecurity. Int. Stud. Manag. Organ..

[50] De Cuyper N., Mäkikangas A., Kinnunen U., Mauno S., De Witte H. (2012). Cross-lagged associations between perceived external employability, job insecurity, and exhaustion: Testing gain and loss spirals according to the Conservation of Resources. Theory J. Organ. Behav..

[51] De Witte H., Pienaar J., De Cuyper N. (2016). Review of 30 years of longitudinal studies on the association between job insecurity and health and well-being: Is there causal evidence?. Aust. Psychol..

[52] Hellgren J., Sverke M. (2003). Does job insecurity lead to impaired well-being or vice versa? Estimation of cross-lagged effects using latent variable modelling. J. Organ. Behav..

[53] Kinnunen U., Mauno S., Siltaloppi M. (2010). Job insecurity, recovery and well-being at work: Recovery experiences as moderators. Econ. Ind. Democr..

[54] Nikolova I., van Dam K., Van Ruysseveldt J., De Witte H. (2019). Feeling weary? Feeling insecure? Are all workplace changes bad news?. Int. J. Environ. Res. Public Health.

[55] Baillien E., De Witte H. (2009). Why is Organizational Change Related to Workplace Bullying?. Role Conflict and Job Insecurity as Mediators. Econ. Ind. Democr..

[56] Rafferty A.E., Griffin M.A. (2006). Perceptions of organizational change: A stress and coping perspective. J. Appl. Psychol..

[57] Lazarus R.S., Folkman S. (1984). Stress Appraisal and Coping.

[58] Alışkan N., Özkoç A.G. (2020). Organizational change and job insecurity: The moderating role of employability. Int. J. Contemp. Hosp. Manag..

[59] Allen J., Jimmieson N.L., Bordia P., Irmer B.E. (2007). Uncertainty during Organizational Change: Managing Perceptions through Communication. J. Chang. Manag..

[60] Arnold A., Staffelbach B. (2012). Perceived post-restructuring job insecurity: The impact of employees’ trust in one’s employer and perceived employability. Ger. J. Hum. Resour. Man..

[61] Keim A.C., Landis R.S., Pierce C.A., Earnest D.R. (2014). Why do employees worry about their jobs? A meta-analytic review of predictors of job insecurity. J. Occup. Health Psychol..

[62] Hair J.F., Black W.C., Babin B.J., Anderson R.E. (2009). Multivariate Data Analysis.

[63] Demerouti E., Bakker A.B. (2008). The Oldenburg Burnout Inventory: A Good Alternative to Measure Burnout (and Engagement). Handbook of Stress and Burnout in Health Care.

[64] Demerouti E., Bakker A., Nachreiner F., Ebbinghaus M. (2002). From mental strain to burnout. Eur. J. Work. Organ. Psychol..

[65] Centefelli R.T., Bassellier G. (2009). Interpretation of Formative Measurement in Information System Research. MIS Quart..

[66] Fleuren B.P., van Amelsvoort L.G., Zijlstra F.R., de Grip A., Kant I. (2018). Handling the reflective-formative measurement conundrum: A practical illustration based on sustainable employability. J. Clin. Epidemiol..

[67] Brondino M., Bazzoli A., Elst T.V., De Witte H., Pasini M. (2020). Validation and measurement invariance of the multidimensional qualitative job insecurity scale. Qual. Quant..

[68] Le Blanc P., De Jonge J., Schaufeli W.B., Chmiel N. (2000). Job stress and health. Introduction to Work and Organizational Psychology: A European Perspective.

[69] Bono J.E., McNamara G. (2011). From the editors: Publishing in “AMJ”–Part 2: Research design. Acad. Manag. J..

[70] Burke R.J., Ng E.S., Wolpin J. (2015). Economic austerity and healthcare restructuring: Correlates and consequences of nursing job insecurity. Int. J. Hum. Resour. Manag..

[71] Nunnally J.C., Bernstein I.H. (1994). Psychometric Theory.

[72] Pallant J. (2005). SPSS Survival Manual: A Step by Step Guide to Data Analysis Using SPSS for Windows.

[73] Hayes A.F. (2009). Beyond Baron and Kenny: Statistical Mediation Analysis in the New Millennium. Commun. Monogr..

[74] Davidson R., MacKinnon J.G. (2000). Bootstrap tests: How many bootstraps?. Econom. Rev..

[75] Field A. (2013). Discovering Statistics Using IBM SPSS Statistics.

[76] Lepine J.A., Podsakoff N.P., Lepine M.A. (2005). A Meta-Analytic Test of the Challenge Stressor–Hindrance Stressor Framework: An Explanation for Inconsistent Relationships Among Stressors and Performance. Acad. Manag. J..

[77] De Witte H., Vander Elst T., De Cuyper N., Vuori J., Blonk R., Price R. (2015). Job insecurity, health and well-being. Sustainable Working Lives.

[78] Vander Elst T., Baillien E., De Cuyper N., De Witte H. (2010). The role of organizational communication and participation in reducing job insecurity and its negative association with work-related well-being. Econ. Ind. Democr..

